# Lasing at topological edge states in a photonic crystal L3 nanocavity dimer array

**DOI:** 10.1038/s41377-019-0149-7

**Published:** 2019-04-24

**Authors:** Changhyun Han, Myungjae Lee, Ségolène Callard, Christian Seassal, Heonsu Jeon

**Affiliations:** 1 Department of Physics and Astronomy, Seoul National University, Seoul, 08826 Republic of Korea; 2 Inter-University Semiconductor Research Centre, Seoul National University, Seoul, 08826 Republic of Korea; 3 Université de Lyon, Institut des Nanotechnologies de Lyon-INL, UMR CNRS 5270, CNRS, Ecole Centrale de Lyon, 69134 Ecully, France; 4 Institute of Applied Physics, Seoul National University, Seoul, 08826 Republic of Korea

**Keywords:** Photonic crystals, Nanophotonics and plasmonics

## Abstract

Topological photonics have provided new insights for the manipulation of light. Analogous to electrons in topological insulators, photons travelling through the surface of a topological photonic structure or the interface of two photonic structures with different topological phases are free from backscattering caused by structural imperfections or disorder. This exotic nature of the topological edge state (TES) is truly beneficial for nanophotonic devices that suffer from structural irregularities generated during device fabrication. Although various topological states and device concepts have been demonstrated in photonic systems, lasers based on a topological photonic crystal (PhC) cavity array with a wavelength-scale modal volume have not been explored. We investigated TESs in a PhC nanocavity array in the Su–Schrieffer–Heeger model. Upon optical excitation, the topological PhC cavity array realised using an InP-based multiple-quantum-well epilayer spontaneously exhibits lasing peaks at the topological edge and bulk states. TES characteristics, including the modal robustness caused by immunity to scattering, are confirmed from the emission spectra and near-field imaging and by theoretical simulations and calculations.

## Shrinking topological state lasers

Tiny lasers that emit light from single nano-sized cavities are becoming possible thanks to exotic photonic features called topological edge states (TESs) on the edges of a cavity array. TESs can steer neatly around obstacles without scattering out; this makes them ideal for navigating the inevitable imperfections that appear when fabricating nanostructures. Heonsu Jeon and co-workers at Seoul National University, Republic of Korea, and Université de Lyon, France, etched patterns of holes a few hundred nanometres apart in photoluminescent wafers, then directed a 1064-nanometre laser to optically excite their sample. They observed an isolated peak of stimulated emission around 1500 nanometres from one cavity, attributed to a TES. This evidence of lasing from sources even smaller than the wavelength of light could enable further minimising of photonic devices and have applications for quantum computing.

## Introduction

Topological insulators, an emerging field in condensed matter physics, are a fascinating research subject owing to their intriguing properties based on coexisting insulating bulk and conducting surface states^1^. Moreover, the conducting surfaces are topologically protected, thereby resulting in immunity to backscattering owing to imperfections or disorder, which makes topological insulators exceptionally valuable from the point-of-view of applications^1^. Recently, various concepts in topological insulators have been adopted and exploited in photonics, which have given rise to a paradigm shift regarding our views on light manipulation, and have led to the emergence of *topological photonics*^2,3^. Seminal work that has triggered topological photonics should be that for unidirectional optical waveguides with broken time-reversal symmetry^4,5^. While the initial demonstration was implemented in a magneto–optical photonic crystal (PhC) in the microwave regime^6^, a recent trend is to realise topological phases in the optical frequency regime without the need for time-reversal symmetry breaking^7–13^. In particular, topological edge states (TESs) should significantly improve the functional robustness of resultant photonic devices owing to their intrinsic immunity against structural imperfections and irregularities, which may be created during device fabrication, making topological photonics a promising and powerful technology of the future. In fact, the race for exotic lasers based on TESs, such as nonreciprocal one-way lasers^14–16^, has already begun.

As for topological structures capable of possessing TESs, the Su–Schrieffer–Heeger (SSH) model, originally introduced to describe linear conjugated polymers^17^, is of supreme importance. Because of its structural simplicity, the photonic SSH model has been extensively studied, and associated TESs have been demonstrated in various platforms, including photonic waveguides^18–23^, plasmonic waveguides^24^, and resonator chains^25–27^. TES lasers based on the SSH model structure have also been demonstrated using micro-pillar arrays^28^, micro-disk arrays^29,30^, and a Fabry-Perot cavity array^31^. However, the unit feature size of these structures is relatively large (~ 10 μm), and their miniaturisation, high-density integration, and performance improvements are thus limited. In this context, an array of PhC nanocavities should be an ideal platform for future TES-based photonic devices because they can offer an extremely small modal volume and high-quality factors^32–34^.

Here, we show both theoretically and experimentally that robust TESs are formed in a finite chain of coupled PhC nanocavities with topologically correct terminations. We implement the structure by arranging identical PhC nanocavities in the SSH dimer chain configuration within a two-dimensional (2D) PhC backbone composed of an InAsP/InP multiple-quantum-well (MQW) epilayer and demonstrate lasing action at the associated TESs (as well as bulk states). Compared with the recent demonstration of TES lasing in a one-dimensional (1D) PhC SSH nanobeam structure^35^, our structures are composed of high-Q nanocavities and are, therefore, closer to the ideal SSH structure where only the nearest-neighbour interaction is concerned, which greatly facilitates the controls of TES properties through structural modifications. In addition, nanocavities within a 2D PhC platform offer a large degree of design flexibility. For example, our device platform can be up-scaled in cavity size (for higher laser output) and reconfigured in 2D space (for better design flexibility and device functionality). Furthermore, the existence and robustness of TESs are proven not only with spectral analyses but also by direct visualisation of the corresponding modal patterns using a near-field optical microscopy technique.

## Results

### Band structure of the infinite PhC nanocavity dimer array

**Fig. 1:**
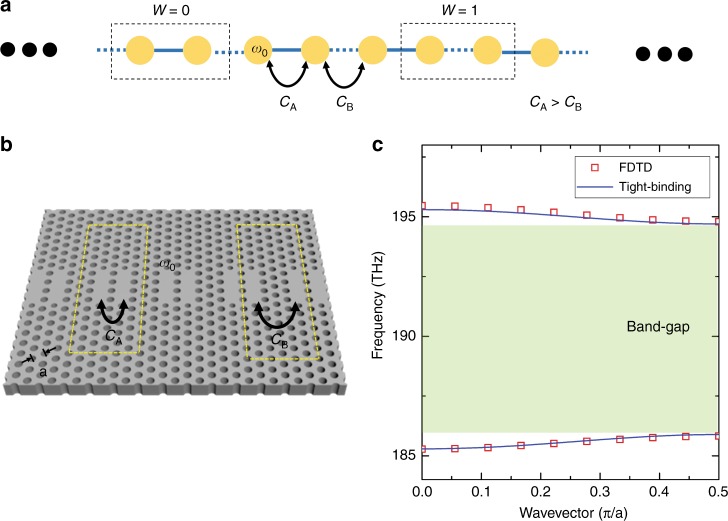
Photonic Su–Schrieffer–Heeger (SSH) structure. **a** Schematic one-dimensional (1D) SSH structure composed of a linear chain of dimerised unit cells. There are two possible choices for the unit cell depending on the winding number: *W* = 0 or 1. **b** The 1D SSH model structure implemented in a 2D hexagonal PhC platform. It was composed of a linear chain of PhC L3 cavities with staggered coupling strengths *C*_A_ and *C*_B_ (*C*_A_ > *C*_B_). **c** Photonic band structure of the PhC-based SSH model structure shown in **b**, calculated by both finite-difference time-domain (FDTD; red open squares) and tight-binding methods (blue solid lines)

In this study, we used a standard SSH model structure, which is depicted in Fig. 1a. It is a 1D chain composed of identical resonators (resonant frequency *ω*_0_) with staggered nearest-neighbour coupling strengths: *C*_1_ = *C*_3_ = ··· = *C*_odd_ = *C*_A_ and *C*_2_ = *C*_4_ = ··· = *C*_even_ = *C*_B_ (*C*_A _> *C*_B_). We realise this structure in a hexagonal lattice PhC slab by linearly placing identical L3 nanocavities with alternating inter-cavity distances of one and three lattice constants, as shown in Fig. 1b. The lattice constant, hole radius, and slab thickness are set to be *a* = 430 nm, *r* = 0.3*a*, and *t* = 230 nm, respectively. Corresponding photonic band structures are calculated using a unit cell composed of two adjacent resonators. The results obtained by two different methods, namely, finite-difference time-domain (FDTD) simulations and tight-binding calculations (see Supplementary Information for the tight-binding calculation formalism), are shown in Fig. 1c. Parameters needed for the tight-binding calculation, such as the resonant frequency and coupling strengths, are deduced from independent FDTD simulations on single and coupled L3 nanocavities. The agreement between the two independent calculation results is excellent. The two sets of outcomes exhibit two energetically symmetric bands separated by a band-gap with a magnitude of 2ǀ*C*_A_ − *C*_B_ǀ. Note that the two possible choices for the dimer unit cell—the dashed boxes in Fig. 1a, b result in the same band structure because we consider here an infinite array.

### Eigenstates for finite PhC nanocavity dimer arrays

**Fig. 2:**
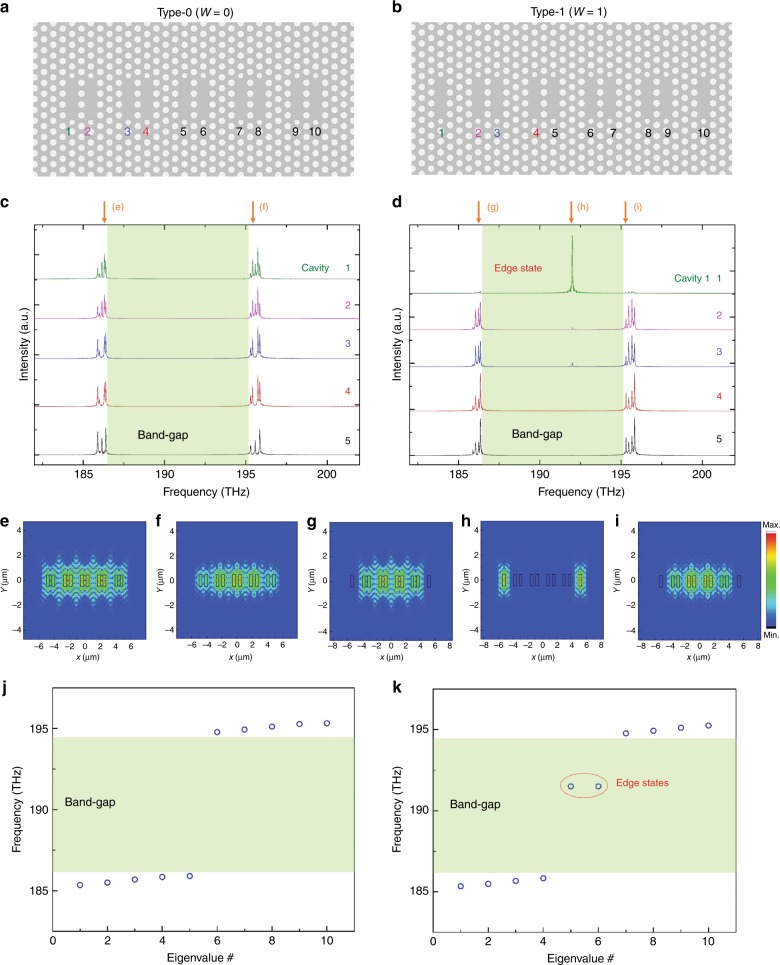
Finite photonic Su–Schrieffer–Heeger (SSH) structures. **a**, **b** Type-0 and Type-1 photonic SSH structures, each composed of ten PhC L3 cavities. The serial numbers 1–10 identify the ten cavities. **c**, **d** Finite-difference time-domain (FDTD)-simulated spectra captured by the monitors set at the cavities of 1 through 5 for Type-0 and Type-1, respectively. **e**, **f** Simulated field profiles of Type-0 at frequencies of (from left) 186.39 and 195.57 THz, respectively. **g**–**i** Simulated field profiles of Type-1 at frequencies of (from left) 186.36, 191.99, and 195.30 THz, respectively. **j**, **k** Ten eigenvalues for the Type-0 and Type-1 cavities, respectively, obtained based on tight-binding calculations

Conversely, for a finite structure, the choice of dimer unit cells is amply important as topological phases at the structural ends become very different. A dimer unit cell can be represented by the winding number *W*^36^, which is defined by

$$W = \frac{{\mathrm{i}}}{{\mathrm{\pi }}}{\oint}_{BZ} {dk\left\langle {u_k\left| {\frac{\partial }{{\partial k}}} \right|u_k} \right\rangle ,}$$

where *u*_*k*_ is a wavefunction of the system described in momentum space *k*. For the unit cell of the two resonators connected by the stronger coupling *C*_A_, the winding number is calculated to be *W* = 0—a trivial topological phase. Conversely, *W* = 1 for the other unit cell as it is intermediated by the weaker coupling *C*_B_, thus indicating a nontrivial topological phase that induces TESs^36^. Figure 2a, b show the two types of cavity arrays considered in this study with the respective winding numbers of *W* = 0 and *W* = 1. Both cavity arrays are composed of ten identical PhC L3 cavities but with different unit cells (or different edge terminations); hereafter, we refer to them as Type-0 and Type-1, respectively, based on their winding numbers.

We performed full three-dimensional FDTD simulations to identify all possible resonant modes supported by the structures. Figure 2c, d display simulated spectra for Type-0 and Type-1 arrays, respectively. Each spectrum in the figures (with a designated cavity number in the range of 1 to 5) was recorded by a monitor set at the centre of the corresponding cavity. Spectra for the remaining cavities (from 6 to 10) are omitted because of the structural symmetry. The most extraordinary spectral feature that distinguishes the two types of PhC nanocavity arrays is that Type-1 exhibits an additional mode inside the band-gap, which was identified as the free (featureless) spectral region between the two bunches of sharp peaks. It is interesting to note that the mode inside the band-gap appeared only at the structural edge (or Cavity 1), whereas the other modes are spatially spread over all the cavities (except for Cavity 1 in Type-1). Therefore, we can speculate that the mode inside the band-gap is a TES, while the other modes are bulk states. As a direct confirmation of this effect, the electric field profiles calculated at a few representative modal frequencies are shown in Fig. 2e–i. For Type-0, the field profiles extend over the entire set of cavities (from Cavity 1 to 10), regardless of the resonant frequencies, as shown in Fig. 2e, f. Conversely, for Type-1, the mode inside the band-gap is strongly localised only at the two end cavities (Cavities 1 and 10), as shown in Fig. 2h, whereas the other modes are spread out (from Cavity 2 to 9), as shown in Fig. 2g, i (see Supplementary Information for the energy distribution among different cavities). These field profiles perfectly match with the simulated spectra in Fig. 2c, d, thus supporting our notion that the mode inside the band-gap is indeed a TES, while the other modes are bulk states.

For an analytical check, a tight-binding model calculation was also performed. The eigenvalues obtained based on this calculation are plotted in Fig. 2j, k for Type-0 and Type-1, respectively. As expected, doubly degenerate TESs appear inside the band-gap for Type-1. All the other eigenstates in both types of nanocavity arrays are outside the band-gap. These observations are consistent with the previous FDTD simulation results in Fig. 2c, d. It is interesting to note that the TES eigenvalues are not exactly in the middle of the band-gap but are shifted to the high-frequency side, which is against the chiral symmetry requirement of the SSH Hamiltonian^22^. In fact, this shift is also observed in the FDTD simulation results, as shown in Fig. 2d. This shift is caused by the simple fact that the mean resonance frequency of the supermodes in coupled cavities changes from that of an uncoupled system. This coupling-induced resonance frequency shift, which often occurs in small coupled cavities, such as PhC nanobeam cavities^37^, modifies the diagonal components of the SSH Hamiltonian and breaks the chiral symmetric spectrum (see Supplementary Information).

### Lasing at topological states

**Fig. 3:**
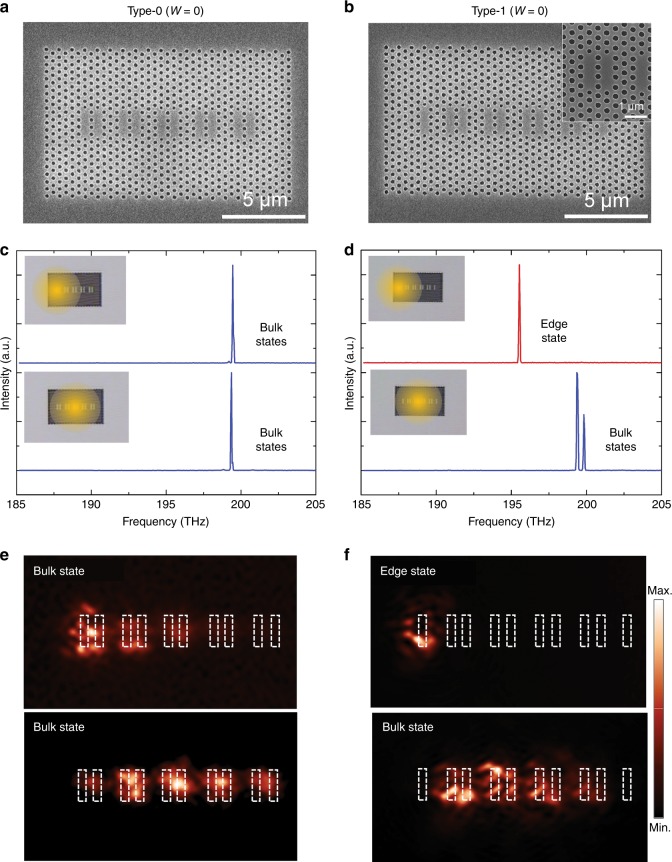
Implementation and characterisation of the finite photonic Su–Schrieffer–Heeger (SSH) structure. **a**, **b** Scanning electron microscopy (SEM) images of the photonic SSH structures with Type-0 and Type-1 cavities fabricated in an InAsP/InP MQW epilayer. The inset in **b** shows a magnified SEM image. **c**, **d** Micro-photoluminescence spectra obtained from structure Type-0 and Type-1, respectively, with the excitation spot centred at the left edge (top panels) and at the centre (bottom panels) of the device. The insets are used to show the positions of the excitation spot (represented by the yellow-shaded circle) relative to the PhC nanocavity arrays. **e**, **f** Near-field scanning optical microscope (NSOM) images recorded for structure Type-0 and Type-1, respectively. The four NSOM images were measured at the corresponding peak emission wavelengths in **c**, **d**

For experimental verification, we realised the proposed PhC nanocavity array structures using an InAsP/InP MQW epistructure bonded onto a transparent substrate. Detailed fabrication steps are described in the Methods Section. Figure 3a, b show scanning electron microscopy (SEM) images of the Type-0 and Type-1 fabricated devices, respectively. As can be observed in the inset of Fig. 3b, the two end-holes in each L3 nanocavity are intentionally shifted in position and decreased in size to reduce the radiative loss^32^. The devices lased upon optical excitation and revealed their own eigenmodes with a high-spectral precision. We intentionally restricted the excitation spot size to ~ 10 μm in diameter so that it did not fully cover the devices (~ 12 μm). In this way, we can have direct control on the type of eigenmode(s) that lases between TESs and bulk states. Figure 3c, d show typical emission spectra obtained from devices of Type-0 and Type-1, respectively, when the excitation spot is located at an edge (top) and the centre (bottom) of an individual device (see the insets for the relative locations of the excitation spot). Interestingly, the lasing spectrum from Type-0 is not sensitive to the location of the excitation spot, as shown in Fig. 3c. This is in contrast with the situation for Type-1 in which the lasing wavelength and the number of lasing modes are changed substantially, as indicated by Fig. 3d. From the spatial correlation between the excitation position (Fig. 3d) and the modal profiles (Fig. 2g–i), we can speculate that the lasing peak induced by the edge excitation of Type-1 (red curve in Fig. 3d) originated from a TES.

To obtain direct evidence for lasing at a TES, we conducted near-field scanning optical microscope (NSOM) measurements, which we have used to directly visualise the spatial field distribution patterns of the lasing modes^38^. Figure 3e, f are NSOM images measured for the lasing peaks respectively shown in Fig. 3c, d. The lasing mode that we speculate to be a TES has a field distribution pattern that is tightly localised at the last cavity (Cavity 1). This is in contrast to the other three lasing modes, which exhibit extended modal patterns across several cavities, indicative of bulk states.

### Robustness of TES lasing

The most important and valuable virtue of TESs from the point-of-view of applications should be the modal robustness. Chiral symmetry in the SSH Hamiltonian is preserved even in the presence of structural perturbations, such as disorder in the coupling strength, so that the modal properties of TESs are hardly altered^22,36^. It is also shown that the edge localisation can be maintained even if the chiral symmetry is broken, such as, for example, by a direct modification of the edge site itself^28^. We investigated the robustness of our TESs against structural fluctuations generated during device fabrication. Below, we present the contrast between the fragility of coupled nanocavities and the robustness of the TES.

**Fig. 4:**
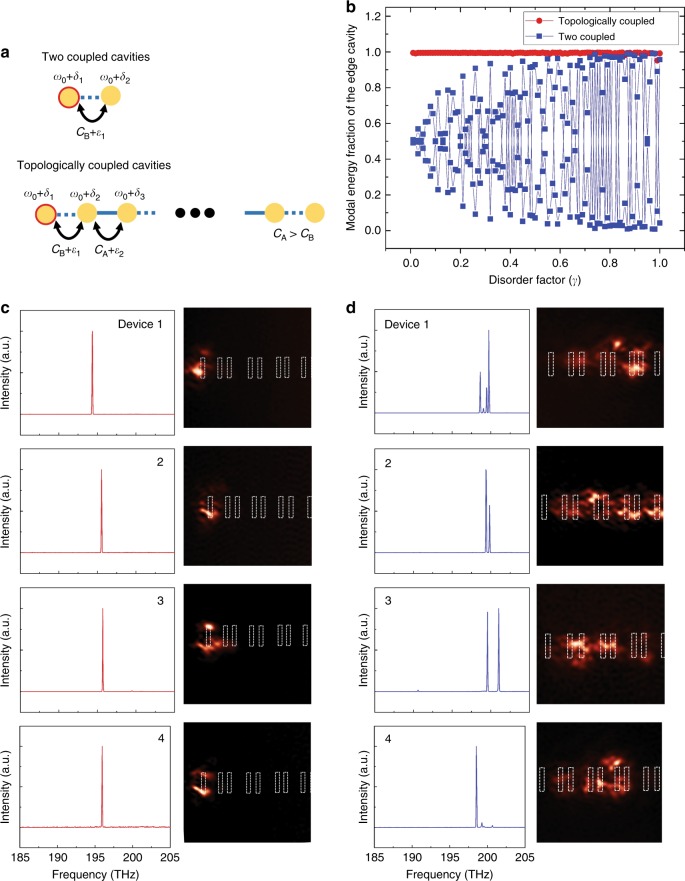
Modal robustness of a topological edge state (TES). **a** Schematics of two coupled cavities (top) and a topologically coupled cavity array (or Type-1, bottom). **b** Electromagnetic energy fraction stored in the first cavity on the left (marked by coloured circles in **a**) as a function of the disorder factor for the coupled two-cavity system (blue squares) and Type-1 coupled cavity array (red circles). **c** Edge-state laser-emission spectra (left) and corresponding near-field scanning optical microscope (NSOM) images (right) acquired from four nominally identical but practically different Type-1 devices. During the measurements, the excitation spot was intentionally shifted to the left to trigger lasing at a TES. **d** Bulk-state laser-emission spectra (left) and corresponding NSOM images (right) from the four Type-1 devices shown in **c** recorded with the excitation spot aimed at the centre of the device to trigger bulk-state lasing

The two model structures to be investigated theoretically are depicted in Fig. 4a: an isolated coupled cavity system (or the unit cell of our Type-1 cavity array) and a topologically coupled cavity array (or the Type-1 cavity array itself). Herein, we allow the occurrence of random fluctuations in the resonant frequency (*ω*_0_ + *δ*_*i*_; *i* = 1, ···, 10) and the coupling strength between cavities (*C*_A/B_ + *ε*_*i*_; *i* = 1, ···, 9). Based on our own experience in semiconductor fabrication, as well as the literature^39,40^, we limit the fluctuations in the resonant frequency to levels below 1% and those in the coupling strength to below 10%. The conditions can be formulated as *δ*_*i*_ = 0.01*γξ*_*i*_ *ω*_0_ and *ε*_*i*_ = 0.1*γξ*_*i*_*C*_A/B_, where *γ* (0 < *γ* <1) is the fluctuation factor that determines the overall degree of fluctuations and *ξ*_*i*_ is a random number between −1 and +1. The tight-binding calculation results for the energy parts stored in Cavity 1 for both structures are shown in Fig. 4b. It is clear that the stored energy in the isolated coupled cavity system fluctuates considerably, regardless of the degree of disorder (*γ*). For the topologically coupled cavity array system, however, it remains >0.95 for the entire range of *γ*, thus implying that the edge mode is hardly affected by structural disorder.

To assess the robustness of the TES experimentally, we examined a group of Type-1 devices that differed from one another only according to the fabrication tolerance. Figure 4c, d show the emission spectra recorded from four Type-1 devices, with the excitation spot focused on an edge and on the centre of the device, respectively. A corresponding NSOM image is also shown in the right side of each spectrum. The four devices are nominally identical as they are not only of the same structural design but are also grown and fabricated in a batch. As observed in Fig. 4c, the nature of single-mode lasing is well preserved with a minor drift in the lasing wavelength (owing to inevitable structural fluctuations). The corresponding NSOM images indicate that lasing indeed originates from a localised TES at the end of the cavity. In contrast, the emission spectra shown in Fig. 4d, which are acquired with the excitation spot aimed at the centre of the nanocavity array, exhibit multiple lasing peaks, with their number, spectral positions, and relative intensity strengths fluctuating considerably, which is a manifestation of typical characteristics for bulk states. The associated NSOM images shown in Fig. 4d, each captured at the wavelength of the highest peak of the corresponding device, show spatially extended modal patterns, thus confirming visually that they belong to the bulk states.

### TES in a kinked nanocavity array

**Fig. 5:**
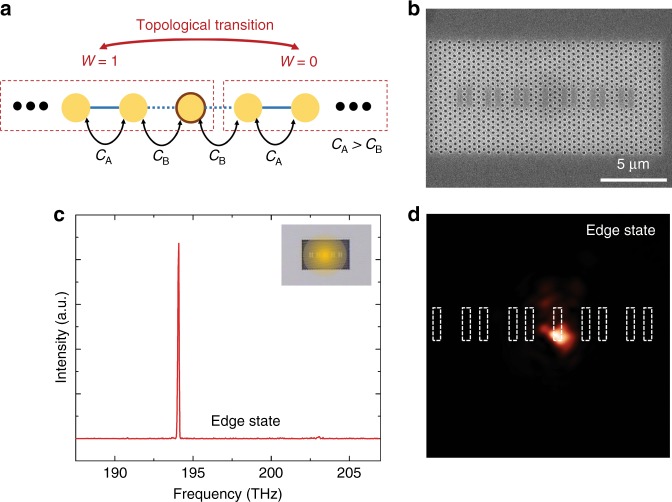
Topological edge state (TES) at a kink. **a** Schematic of a photonic Su–Schrieffer–Heeger (SSH) structure with a kink in the middle. The structure can be viewed as two separate SSH structures with different winding numbers that are attached to each other side-by-side. **b** Scanning electron microscopy (SEM) image of a fabricated PhC-based SSH structure with a kink. **c** Micro-photoluminescence spectrum from a fabricated device with the excitation spot at the kink. **d** Near-field scanning optical microscope (NSOM) image recorded at the lasing wavelength shown in **c**

An alternative way of creating a TES is to insert a kink in the middle of a coupled cavity array. A kink in a dimerised cavity array is equivalent to two dimer arrays of different topological invariants placed side by side, as shown in Fig. 5a. A single edge-state then appears at the interface of the two dimer arrays owing to the bulk-edge correspondence^1^. To verify this, we fabricated another PhC L3 nanocavity array with an intentional kink in the middle, as shown by the SEM image in Fig. 5b. When the centre of the nanocavity array was excited, we readily observed a single-mode lasing peak, as shown in Fig. 5c. The NSOM examination revealed that the single-mode lasing is indeed from a mode that is tightly localised at the kink where the two nanocavity arrays with different topologies meet, as shown in Fig. 5d. This demonstration is a proof of the design flexibility of sophisticated and intriguing TES-based photonic devices.

## Discussion

We have demonstrated both theoretically and experimentally the existence of TESs in a 1D dimer chain composed of PhC L3 nanocavities. FDTD simulations applied to the model structures unambiguously showed that TESs could be formed on the wavelength scale, depending on the edge type of the nanocavity array. We implemented the designed structures using an InAsP/InP MQW epilayer. Upon optical excitation, TESs were materialised spontaneously as a sharp lasing peak. This implies that TESs possess cavity Q-factors that are adequately high for photonic device applications. We directly visualised the nature of the edge states (as well as the bulk states) by measuring modal field profiles using the NSOM technique. In addition, we demonstrated the robustness of the TESs against structural fluctuations, which may be the most important virtue of TESs compared to ordinary PhC nanocavities from an applications point-of-view.

Our results prove unambiguously that TESs in a PhC SSH structure can serve as wavelength-scale nanocavities that possess adequately high Q-factors for lasing while simultaneously presenting immunity to structural irregularities. These properties are of high-technological value, as well as practical importance for robust photonic devices for exotic functions, such as quantum information^41^, nonlinear light generation^42^, and densely integrated photonic systems^43–45^. In particular, strong nonlinear effects and functionalities are expected from TESs because of their high *Q*/*V* ratios. Therefore, the PhC-based SSH structure and associated TESs are not only an intriguing fundamental research object in topological photonics but may also allow the implementation of exotic devices on the nanometre scale.

## Materials and methods

### Device fabrication

The PhC cavity arrays were fabricated on an InAsP/InP MQW epilayer designed to emit at a wavelength of ~ 1550 nm, which was grown epitaxially on an InP substrate. The epi-wafer was flip-bonded to a fused silica substrate using a molecular bonding method. The substrate was then removed by wet chemical etching in an HCl solution. For patterning nanocavity arrays, a silicon-nitride hard-mask layer was deposited at a thickness of ~ 50 nm on the bonded MQW slab (~ 230 nm in thickness) by plasma-enhanced chemical vapour deposition. A positive electron-beam resist (ZEP 520A, Zeon) was spin-coated, and the PhC patterns were generated using an electron-beam lithography system (JBX–6300FS, JEOL). The patterns were sequentially transferred to the silicon-nitride hard-mask layer and to the MQW slab using reactive ion etching (RIE 80 Plus, Oxford Instrument). Finally, the resist and hard-mask layer were removed to complete the fabrication.

### Optical measurements

A pulsed laser diode emitting at 1064 nm was used to optically excite devices. A 50 × objective lens was simultaneously used for the optical excitation of the device and for the collection of light from the device. The sample was mounted on an actuator stage to control the position of the excitation spot. Collected light was sent to an optical spectrum analyser to obtain photoluminescence spectra. Near-field images were obtained using a custom-made NSOM apparatus operating in the near-IR regime. The sample was mounted on an inverted optical microscope (Eclipse Ti-S, Nikon) and was optically excited through the silica substrate (from the backside). Evanescent fields for the lasing modes were captured by a dielectric NSOM probe, while the probe position was manipulated by a scanning controller system (SMENA, NT–MDT). Collected near-field signals were fed to a monochromator equipped with an InGaAs photodiode (MicroHR, Horiba, DSS-IGA025T, Horiba). A lock-in amplifier (SR830, Stanford Research Systems) was used to enhance the signal-to-noise ratio.

### Numerical simulations

Numerical simulations were conducted using commercial FDTD software (FDTD solutions, Lumerical). To excite all possible resonant modes, randomly oriented dipole sources with arbitrary polarisations and phases were placed across the entire simulation space. Fourier-transformed signals collected by a time monitor generated spectral and modal profiles. For band structure calculations, a single unit cell constituted the simulation region with the Bloch boundary condition along the direction of the cavity array. The Bloch wave-vector value was then scanned in a series of simulations.

## Supplementary information

SUPPLEMENTARY INFORMATION for Lasing at topological edge states in a photonic crystal L3 nanocavity dimer array
