# Supplementary material for: Lasing at topological edge states in a photonic crystal L3 nanocavity dimer array
Source: Light Sci Appl. 2019 Apr 24;8:40. doi: 10.1038/s41377-019-0149-7 (PMC6478828; doi:10.1038/s41377-019-0149-7)
Supplement: Supplementary file 1 — SUPPLEMENTARY INFORMATION for Lasing at topological edge states in a photonic crystal L3 nanocavity dimer array [file 41377_2019_149_MOESM1_ESM.docx]

Supplementary Information for

**Lasing at topological edge states in a photonic crystal L3 nanocavity dimer array**

Changhyun Han^1,2^, Myungjae Lee^1,2^, Ségolène Callard^3^, Christian Seassal^3^, and Heonsu Jeon^1,2,4*^

^1^Department of Physics and Astronomy, Seoul National University, Seoul 08826, Republic of Korea

^2^Inter-University Semiconductor Research Centre, Seoul National University, Seoul 08826, Republic of Korea

^3^Université de Lyon; Institut des Nanotechnologies de Lyon-INL, UMR CNRS 5270, CNRS, Ecole Centrale de Lyon, Ecully F-69134, France

^4^Institute of Applied Physics, Seoul National University, Seoul 08826, Republic of Korea

**Tight-binding formalism for band structure calculation**

The Hamiltonian of the SSH model system^1^is given by

$H= \sum_{n} C_{A}(b_{n}^{\dagger}a_{n}+a_{n}^{\dagger}b_{n})+C_{B}\left( b_{n-1}^{\dagger}a_{n}+a_{n-1}^{\dagger}b_{n} \right)+\omega_{0}a_{n}^{\dagger}a_{n}+\omega_{0}b_{n}^{\dagger}b_{n}$,

where $a_{n}$($a_{n}^{\dagger}$) is the annihilation (creation) operator and $C_{A}$($C_{B}$) is the coupling constant of the strong (weak) bond while $\omega_{0}$ is the resonance frequency of each cavity. The operators *a* and *b* denote the two cavities within unit cell. After Fourier transformation, the operators can be expressed as

$a_{n}=\frac{1}{\sqrt{N}}\sum_{k} a_{k}e^{-ik{na}_{0}}$; $b_{n}=\frac{1}{\sqrt{N}}\sum_{k} b_{k}e^{-ik{na}_{0}}$,

where a_0_ is a lattice constant. Then, the Hamiltonian in the momentum space becomes

$$H\left( k \right)= \left( \begin{matrix} \omega_{0} & C_{A}+C_{B}e^{-ika_{0}} \\ C_{A}+C_{B}e^{ika_{0}} & \omega_{0} \end{matrix} \right)$$

Solving for the eigenvalues results in the following dispersion relation:

$$\lambda=\omega_{0}\pm\sqrt{C_{A}^{2}+C_{B}^{2}+2C_{A}C_{B}\cos\left( ka_{0} \right)}$$

By inserting numerical values on the resonant frequency and coupling constants, which are extracted from independent FDTD simulations on single and coupled cavities, we obtain Fig. 1c.

**Winding number calculation for the SSH system**

Starting with the SSH Hamiltonian,

$$H\left( k \right)=\left( \begin{matrix} \omega_{0} & C_{A}+C_{B}e^{-ika_{0}} \\ C_{A}+C_{B}e^{ika_{0}} & \omega_{0} \end{matrix} \right)=C_{B}\left( \begin{matrix} \omega_{0}/C_{B} & C_{A}/C_{B}+e^{-{ika}_{0}} \\ C_{A}/C_{B}+e^{{ika}_{0}} & \omega_{0}/C_{B} \end{matrix} \right),$$

we define

$$\rho\left( k \right)=C_{A}/C_{B}+e^{-ika_{0}}=\left| \rho\left( k \right) \right|e^{-ik\phi(k)},$$

where

$$\phi\left( k \right)=arctan(sin(ka_{0})/(C_{A}/C_{B}+cos \left( ka_{0} \right)).$$

Then the SSH Hamiltonian becomes

$$H\left( k \right)=C_{B}\left( \begin{matrix} \omega_{0}/C_{B} & \left| \rho\left( k \right) \right|e^{-ik\phi\left( k \right)} \\ \left| \rho\left( k \right) \right|e^{ik\phi\left( k \right)} & \omega_{0}/C_{B} \end{matrix} \right),$$

from which we obtain the eigenvectors

$$u\left( k \right)=\frac{1}{\sqrt{2}}\left( \begin{matrix} e^{-i\phi\left( k \right)} \\ \pm1 \end{matrix} \right).$$

The winding number can then be calculated to be

$$W=\frac{i}{\pi}\oint_{BZ} dk\langle u\left( k \right)\left| \frac{\partial}{\partial k} \right|u\left( k \right)\rangle=\frac{1}{2\pi}\oint_{BZ} dk\frac{d\phi\left( k \right)}{dk}=\frac{\Delta\phi}{2\pi},$$

where $\Delta\phi$ is the accumulated phase of $\phi(k)$, as *k* evolves across the Brillouin zone.

From the definition of $\phi(k)$, $\phi(k)$ acquires a full phase of $2\pi$ ($W=1)$ if $C_{A}/C_{B}>1$, and 0 ($W=0)$ if $C_{A}/C_{B}<1$*.*

**Localization of the TES**

The field strength of an edge state is given by an exponential decaying function $\left( \frac{C_{B}}{C_{A}} \right)^{n}=\left( \frac{C_{A}}{C_{B}} \right)^{-n},$ where the exponent stands for the n-th unit cell from the edge^2^. We obtain the field profiles of the TES in our system using both FDTD simulations and tight-binding model calculations, which are consistent with each other as shown in Fig. S1. By fitting the results, we obtain $\sim\left( 14.4 \right)^{-n}$ from the FDTD simulations and $\sim\left( 14.5 \right)^{-n}$ from the tight-binding calculations. The decay rates are slightly slower than $\left( \frac{C_{A}}{C_{B}} \right)^{-n}\approx\left( 15.6 \right)^{-n}$. We attribute these discrepancies to the finiteness of actual structure and also to the coupling-induced resonant frequency shift, the latter being explained in the next section of SI in great detail.


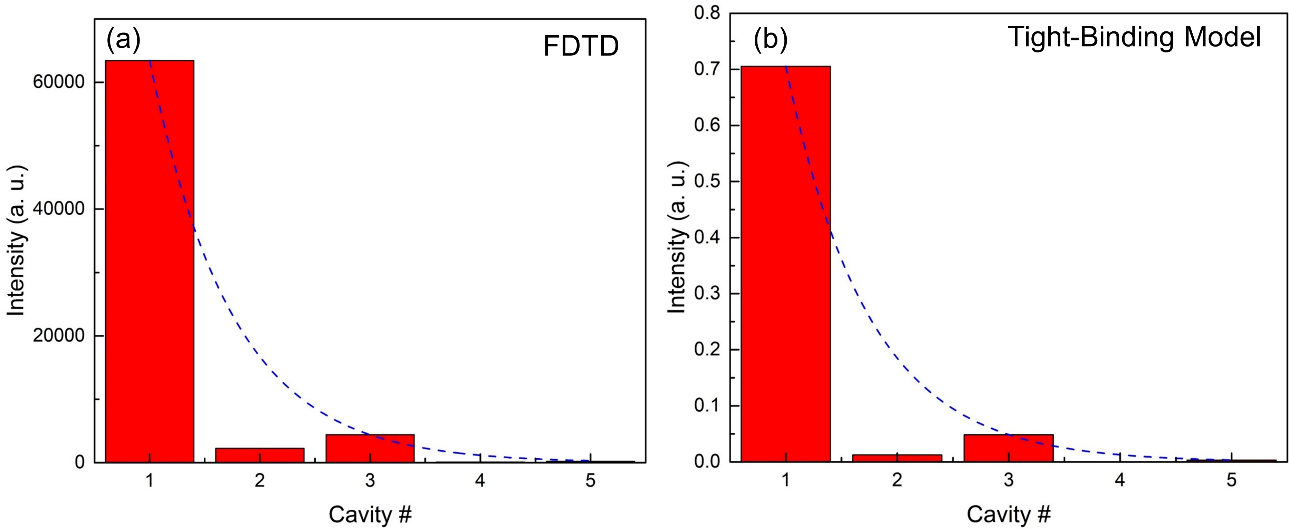


**Figure S1**. Modal amplitudes of the edge state obtained by the FDTD simulation (a) and tight-binding model calculation (b). The dashed lines are exponential fitting curves.

**Coupling-induced resonance shift**


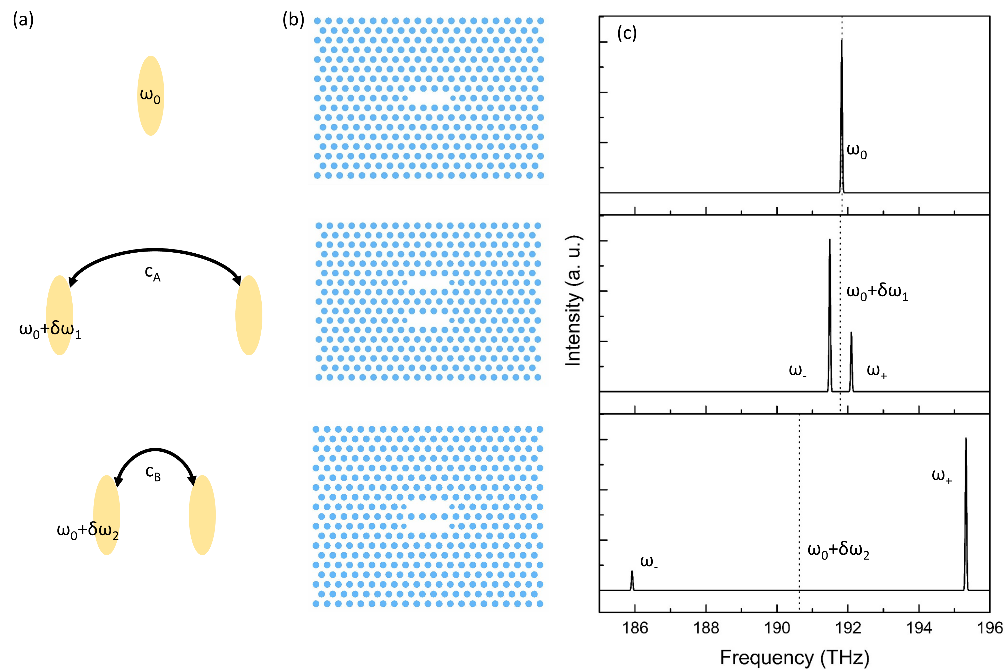
It is known that the mean frequency of the supermodes in coupled resonators (or an *effective* uncoupled resonance frequency) shifts from the single resonator resonance frequency,^3^ which is schematically depicted in Fig. S2(a). It is also known that the amount of shift increases as the coupling becomes stronger. To confirm this, we conducted FDTD simulations on a single PhC L3 cavity and coupled PhC L3 cavities with two different coupling strengths⎯Fig. S2(b). Simulated results, which are shown in Fig. S2(c), clearly demonstrate that the mean value of two resonance frequencies indeed shifts and that the shift gets larger $(\delta\omega_{1}<\delta\omega_{2})$ as the coupling strength is increased.

**Figure S2**. (a) Schematic description of the resonant frequency shift in coupled cavities. (b) Simulation model structures (from top, single L3 cavity and coupled L3 cavities with weak and strong couplings (or three and one lattice constant in terms of the inter-cavity spacing, respectively). (c) Simulated spectra for the corresponding structures in (b).

We then investigate the coupling-induced resonance shift (CIRS) in our topological array system (Type-1). Taking the frequency shift in coupled cavities into account, the SSH Hamiltonian for a finite coupled cavity array system can be rewritten as

$$H=\left( \begin{matrix} \omega_{0}+\delta\omega_{1} & C_{B} & & & \\ C_{B} & \omega_{0}+\delta\omega_{1}+\delta\omega_{2} & C_{A} & & \\ & C_{A} & \ddots& C_{A} & \\ & & C_{A} & \omega_{0}+\delta\omega_{1}+\delta\omega_{2} & C_{B} \\ & & & C_{B} & \omega_{0}+\delta\omega_{1} \end{matrix} \right).$$

Note that the cavities in the middle of the array have two nearest-neighbor cavities on both sides so its effective uncoupled resonant frequency is increased by $\delta\omega_{1}+\delta\omega_{2}$ from the single cavity frequency $\omega_{0}$, while two cavities at the array edges have only one nearest-neighbor so the effective uncoupled resonant frequency is increased by $\delta\omega_{1}$. Diagonalization of the Hamiltonian results in the eigenvalues (resonant frequencies) and corresponding eigenstates. In Fig. S3, we compare results with and without the CIRS included in the Hamiltonian. Without the CIRS, the edge states appear exactly at the center of the band-gap because of the symmetry of the system. On the other hand, if the CIRS is included, the edge states shift from the gap center.


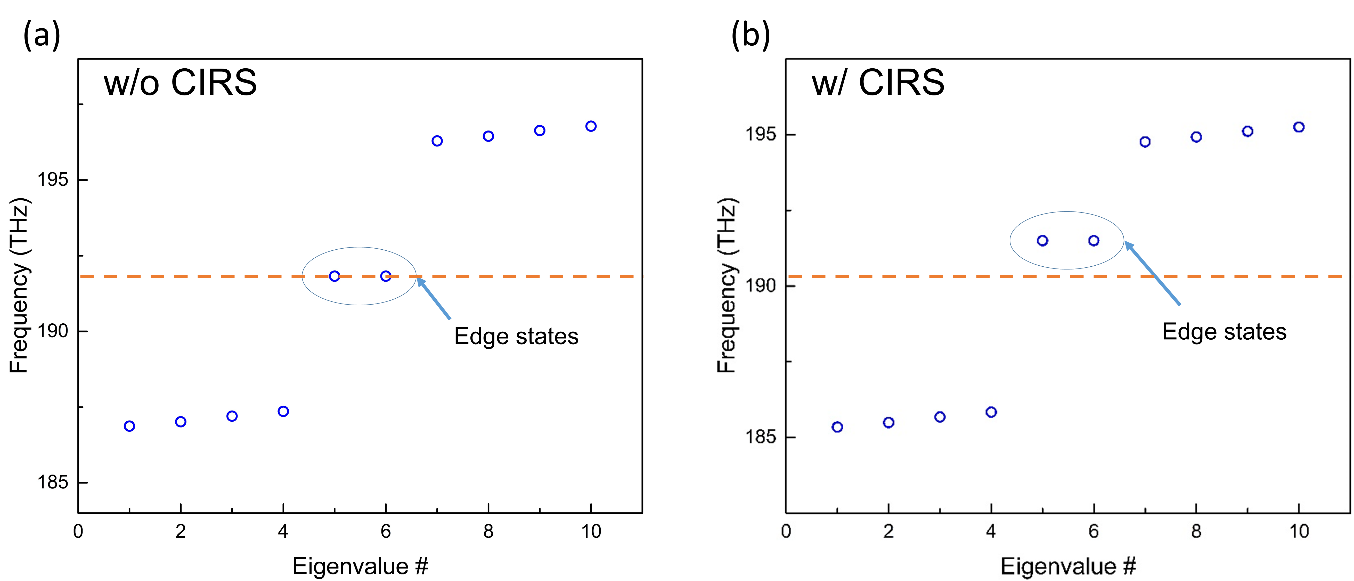


**Figure S3.** Eigenvalues of the SSH model without (a) and with (b) the CIRS.

**Sample fabrication steps**


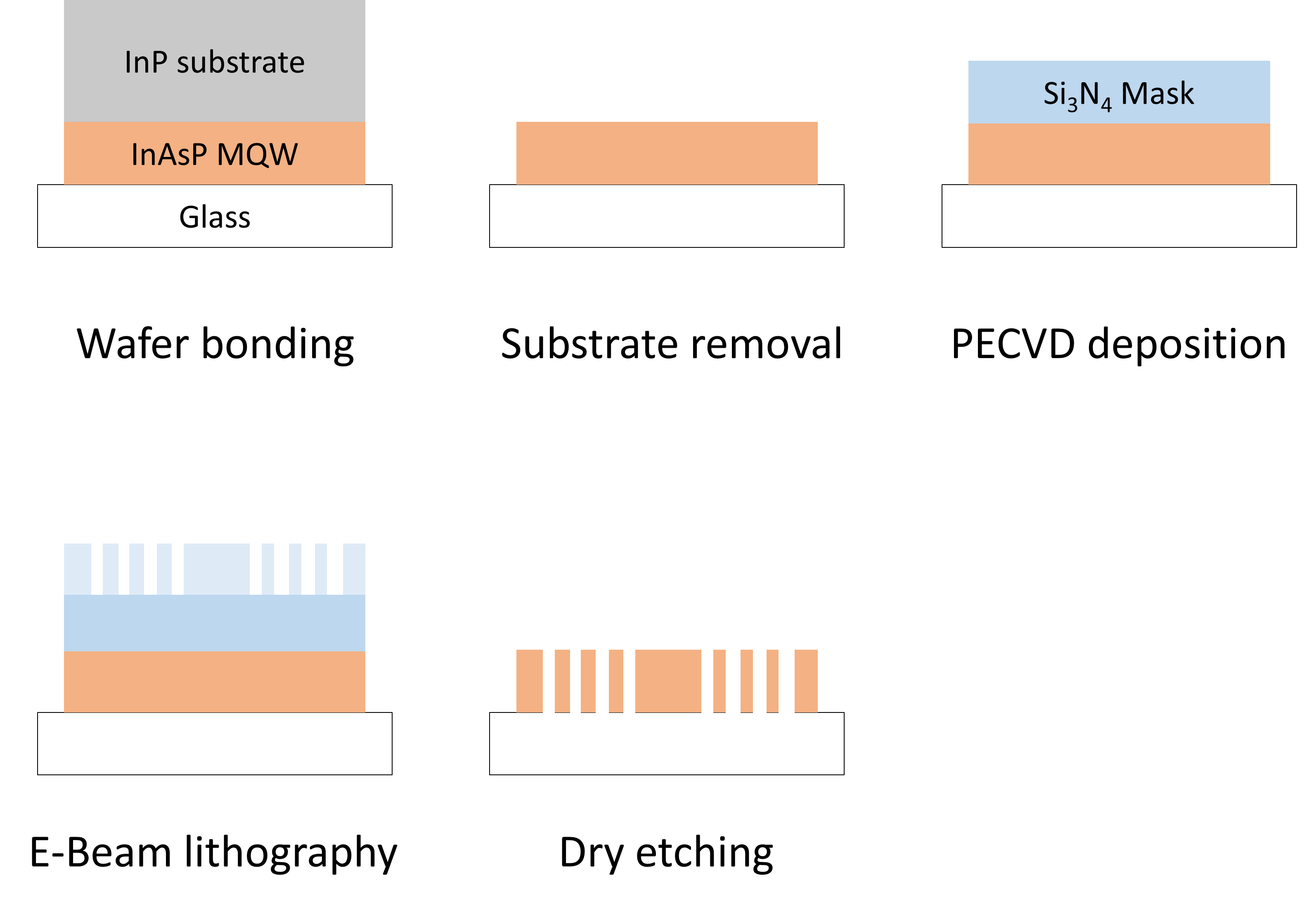


**Figure S4.** Schematic of the device fabrication steps.

**Comparisons between the edge and bulk states on lasing properties**

Presented in Fig. S5 (a) are light-in versus light-out relationships for the edge state and a bulk state measured from a typical Type-1 device. The bulk state shows a higher threshold than the edge state, which we attribute to lower Q-factors of the bulk states and also to gain sharing among multiple bulk states. Q-factors calculated for the Type-1 model structure are plotted in Fig. S5(b), indicating that the Q-factor of the edge state is indeed the highest. Nevertheless, the bulk state exhibits a higher slope efficiency, which we believe is due to a larger modal (or gain) volume occupied by the bulk state.

**
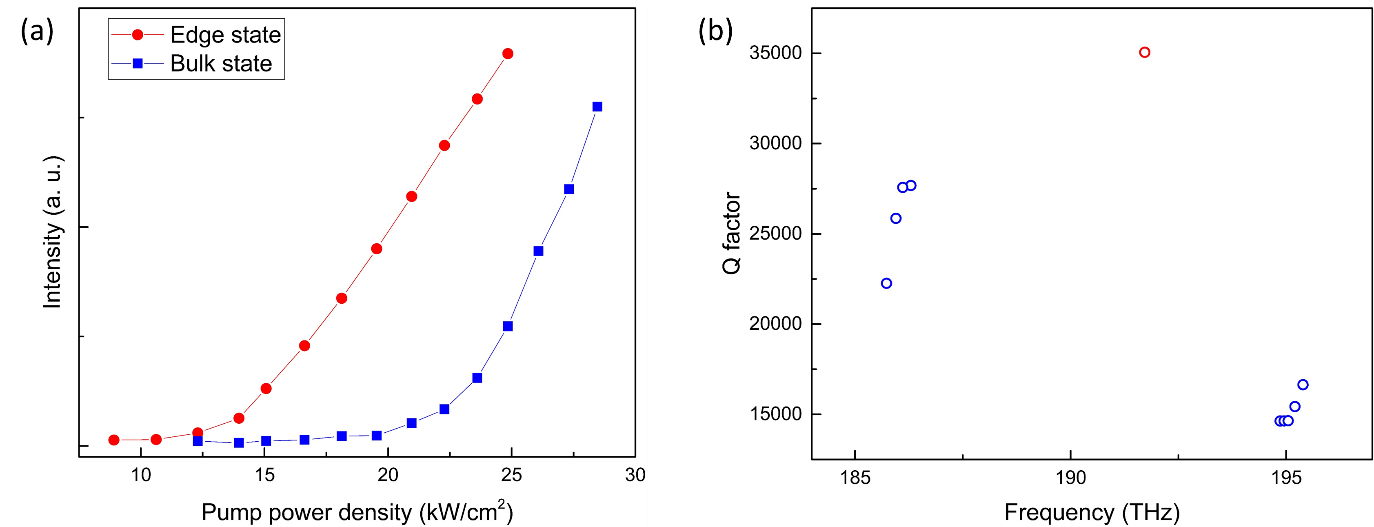
**

**Figure S5.** (a) Light-in versus light-out characteristics of an edge (red dots) and a bulk (blue dots) states, measured from the Type-1 device whose emission spectra are shown in Fig. 3d in the main text. (b) FDTD-simulated Q-factors of the eigenmodes in Type-1 device. The red circle represents the edge state while the blue circles are for the bulk states.

**Estimation of the spontaneous emission factor of the edge state**

To estimate the spontaneous emission factor (*β*) of our TES lasers, we performed a rate equation analysis on the measured L-L curve. High spontaneous emission factor is beneficial for the nanolasers with high Q factor and small modal volume V (or high *Q/V*), an ideal platform to realize thresholdless lasers. The rate equations we consider here are

$$\frac{dN}{dt}=\frac{\eta L_{\mathrm{in}}}{\hbar\omega_{\mathrm{pump}}V_{a}}-\left( \frac{v_{s}}{d_{a}}N+BN^{2}+CN^{3} \right)-\Gamma\frac{g_{0}c}{n_{\mathrm{eff}}}\ln\left( \frac{N}{N_{\mathrm{tr}}} \right)P;$$

$$\frac{dP}{dt}=\Gamma\frac{g_{0}c}{n_{\mathrm{eff}}}\ln\left( \frac{N}{N_{\mathrm{tr}}} \right)P-\frac{P}{\tau_{p}}+\beta BN^{2}.$$

The definitions and values of the parameters are summarized in Table 1. The fitted L-L curve is shown in Fig. S6. The best fitted spontaneous emission factor is estimated to be about 0.15, which is comparable to those of previously reported PhC nanolasers but much better than other topological nanocavity lasers including that in Reference 34.

**Table 1.** Parameters used in rate equation calculation.

| $\eta$(Absorption of pump) | $0.15$ |
| --- | --- |
| $\Gamma$(Confinement factor) | $0.175$ |
| $\tau_{p}$(Photon life time) | $0.3\times{10}^{-9}s^{-1}$ |
| $g_{0}$(Gain coefficient) | $1500 \mathrm{cm}^{-1}$ |
| $B$(Bimolecular recombination rate) | $1.0\times{10}^{-10} cm^{3}s^{-1}$ |
| $C$(Auger recombination rate) | $5.0\times{10}^{-29} \mathrm{cm}^{6} s^{-1}$ |
| $N_{\mathrm{tr}}$(Transparency carrier density) | $1.0\times{10}^{18}cm^{-6}$ |
| $v_{s}$ (Surface recombination velocity) | $3000{cm s}^{-1}$ |
| $d_{a}$ (Propagation distance for surface recombination) | $2.0\times{10}^{-5}\mathrm{cm}$ |
| $n_{\mathrm{eff}}$ (Effective refractive index) | $2.5$ |
| $V_{a}$ (Active volume) | $2.6\times{10}^{-11} \mathrm{cm}^{3}$ |


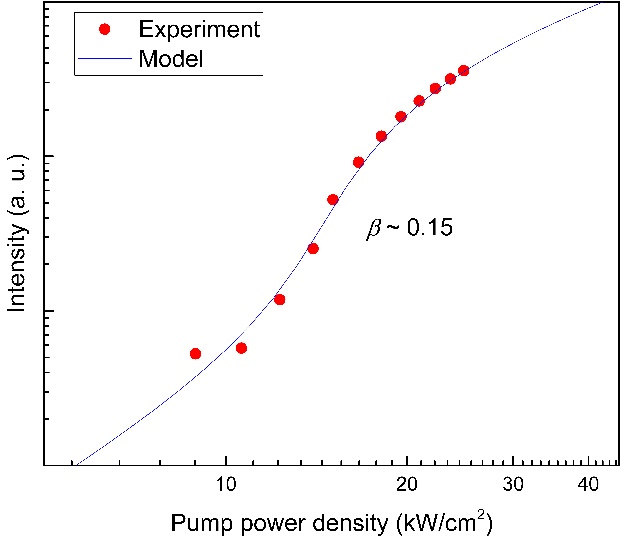


**Figure S6.** Light-in versus light-out curve of our TES laser in the log-log scale. The red dots are experimental data points and the solid line is a fitted curve based on the rate equation calculation.

**Supplementary References**

1 Delplace, P., Ullmo, D. & Montambaux, G. Zak phase and the existence of edge states in graphene. *Phys. Rev. B* **84**, 195452, (2011).

2 St-Jean, P. *et al.* Lasing in topological edge states of a one-dimensional lattice. *Nat. Photon.* **11**, 651-656, (2017).

3 Deotare, P. B., McCutcheon, M. W., Frank, I. W., Khan, M. & Lončar, M. Coupled photonic crystal nanobeam cavities. *Appl. Phys. Lett.* **95**, 031102, (2009).
